# Rua de mão dupla: intercâmbios transatlânticos entre cientistas sociais franceses e brasileiros

**DOI:** 10.1590/S0104-59702026000100007

**Published:** 2026-03-30

**Authors:** Helga da Cunha Gahyva

**Affiliations:** i Professora, Instituto de Filosofia e Ciências Sociais/Universidade Federal do Rio de Janeiro; professora e pesquisadora, Programa de Pós-graduação em Sociologia/Universidade Federal do Rio de Janeiro. Rio de Janeiro – RJ – Brasil. helga.gahyva@gmail.com

Um espectro ronda as nações americanas – o espectro da cópia, como atesta a recorrência, na imaginação social e política específica, mas não exclusivamente nacional, do problema da imitação de ideias, padrões de conduta e instituições estrangeiras. A questão ganhou renovado fôlego, na segunda metade do século XX, via Roberto Schwarz (1987, p.30), cuja discussão sobre o “caráter imitativo da nossa vida cultural” indica que “existe entre as pessoas educadas do Brasil ... o sentimento de viverem entre instituições e ideias que são copiadas do estrangeiro e não refletem a realidade local” (p.38-39). Já Ângela Alonso (2009, p.3) procurou alternativa ao privilegiar elos entre cultura e experiências sociais concretas, deslocando o “problema da imitação para o da apropriação de ideias estrangeiras”.

Ian Merkel (2023, p.16) reconfigura a problemática, elaborando uma “história transnacional das ciências sociais” concentrada nos intercâmbios intelectuais entre cientistas sociais brasileiros e franceses dos anos 1930 aos 1960. Reconhecendo a desigualdade em tais “termos de troca”, ele revela como, a despeito do impacto, no país, dos saberes e práticas gestados no Velho Mundo, é possível explorar o percurso reverso: “Os brasileiros foram parceiros cruciais na reformulação das ciências sociais francesas” (p.22). Seu trabalho inclui-se, assim, entre “abordagens transnacionais que ressaltam a pluralidade de fluxos e de trocas entre diferentes contextos nacionais e ... evitam o paradigma centro-periferia” (Maia, 2019, p.174).

No primeiro capítulo de *Termos de troca: intelectuais brasileiros e as ciências sociais francesas*, Merkel (2023) articula a fundação da Universidade de São Paulo (USP), em 1934, ao fracasso da Revolução Constitucionalista de 1932, quando as elites locais compensaram a redução de seu poder político com investimentos no ensino universitário. Tal contexto contribuiu para definir a feição dos cientistas sociais participantes das missões francesas dirigidas à cidade: em sua maioria em início de carreira, mostraram-se dispostos à remodelação curricular, tanto metodológica quanto empiricamente. Pierre Monbeig, Fernand Braudel, Claude Lévi-Strauss e Roger Bastide protagonizaram esse processo.

O segundo capítulo dedica-se à aclimatação de Monbeig, Braudel e Lévi-Strauss quando aqui aportaram em 1935. Alçados quase instantaneamente à posição de “estabelecidos”, encontraram, em São Paulo, condições para a proposição de uma “ciência social federativa” (Merkel, 2023, p.100).

O capítulo seguinte incorpora Bastide à análise – ele chegou ao país em 1938 – e investiga como os projetos empreendidos pelos quatro pesquisadores contribuíram tanto para lhes abrir chaves de compreensão sobre as ciências sociais quanto para os inserir em uma “rede transatlântica de intelectuais” (Merkel, 2023, p.154).

Durante a Segunda Guerra Mundial, permaneceram no país Monbeig e Bastide; Braudel, na França desde 1937, passou parte do período encarcerado; e Lévi-Strauss migrou para os EUA. Apesar dos obstáculos e deslocamentos, mantiveram intercâmbio com intelectuais brasileiros. Essas trocas “se revelaram cruciais para suas ideias sobre fenômenos como a colonização de povoamento, o sincretismo religioso, o comércio transatlântico e as estruturas de parentesco” (Merkel, 2023, p.169).

Finda a guerra, Bastide continuou no Brasil até 1954, enquanto os demais se instalaram na França. Qualificados, então, para disputar protagonismo em território francês dentro das ciências sociais, mobilizaram, lá e cá, a rede de contatos estabelecida no Novo Mundo, como atestam as articulações para a fundação da sexta seção de École Pratique des Hautes Études.

Isso significou a inserção de temáticas brasileiras em disciplinas e publicações que reforçavam o interesse francês pela América Latina, em geral, e pelo Brasil, particularmente. Ainda sob o trauma da Ocupação, a suposição de que a história brasileira forjara certo padrão de democracia étnica contribuía para a imagem do país, na França, como “um espaço de liberdade e de novas possibilidades” (Merkel, 2023, p.246).

Merkel elege *Tristes trópicos*, de Lévi-Strauss (publicado em 1955), símbolo desse interesse. Nos anos 1950, entretanto, os termos do intercâmbio atingiam dimensões mais igualitárias, pois, além de sua importância como objeto de estudos, o Brasil se constituía como “um sujeito coletivo, localizado nos professores que haviam feito parte das missões francesas para a USP na década de 1930” (Merkel, 2023, p.280).

Braudel e Bastide colaboraram para a notoriedade de Gilberto Freyre entre o público universitário francês durante a primeira metade do século XX. O conceito de “colonialismo esclarecido” (Barbosa, 2023, p.182) sintetiza o modo por meio do qual parcela da intelectualidade se apropriou das ideias do brasileiro: durante a Guerra Fria, a apologia freyriana da miscigenação racial fora mobilizada para conferir verniz humanista ao claudicante império colonial francês.

Por essa época, entretanto, Freyre tornara-se alvo de críticas em seu país natal. Na USP, especificamente, a rotinização do ensino universitário formara cientistas sociais com orientações políticas e metodológicas opostas às suas. Merkel destaca a atuação de Florestan Fernandes, cuja denúncia do “mito da democracia racial” impactara Bastide. Apesar de ter representado o “epicentro da teia de relações em torno da publicação e recepção de *Casa-grande e senzala*” (Barbosa, 2023, p.110) na França, *Relações raciais entre negros e brancos em São Paulo* (publicado em 1955) destaca a existência de discriminação racial na cidade.

A narrativa se encerra nos anos 1960, quando, por um lado, o prestígio de Monbeig, Braudel, Lévi-Strauss e Bastide os tornou mais independentes das redes de relações outrora estabelecidas. Por outro, a consolidação do ensino universitário possibilitou que a recepção da sociologia francesa fosse filtrada pelos pesquisadores brasileiros.

Se, de lá para cá, variaram conjunturas e personagens, permaneceu o intercâmbio entre as tradições nacionais. Assim, Merkel, além de sua contribuição para a história das ciências sociais francesas e brasileiras durante esse período, instiga análises sobre o modo como se recompuseram, desde então, esses termos transnacionais de troca.
